# Foveal Crowding Resolved

**DOI:** 10.1038/s41598-018-27480-4

**Published:** 2018-06-15

**Authors:** Daniel R. Coates, Dennis M. Levi, Phanith Touch, Ramkumar Sabesan

**Affiliations:** 1 College of Optometry, University of Houston, Houston, TX USA; 2 School of Optometry, Vision Science Graduate Group, Helen Wills Neuroscience Institute, University of California, Berkeley, Berkeley, CA USA; 3 Department of Ophthalmology, University of Washington School of Medicine, Seattle, WA USA

## Abstract

Crowding is the substantial interference of neighboring items on target identification. Crowding with letter stimuli has been studied primarily in the visual periphery, with conflicting results for foveal stimuli. While a cortical locus for peripheral crowding is well established (with a large spatial extent up to half of the target eccentricity), disentangling the contributing factors in the fovea is more challenging due to optical limitations. Here, we used adaptive optics (AO) to overcome ocular aberrations and employed high-resolution stimuli to precisely characterize foveal lateral interactions with high-contrast letters flanked by letters. Crowding was present, with a maximal edge-to-edge interference zone of 0.75-1.3 minutes at typical unflanked performance levels. In agreement with earlier foveal contour interaction studies, performance was non-monotonic, revealing a recovery effect with proximal flankers. Modeling revealed that the deleterious effects of flankers can be described by a single function across stimulus sizes when the degradation is expressed as a reduction in sensitivity (expressed in Z-score units). The recovery, however, did not follow this pattern, likely reflecting a separate mechanism. Additional analysis reconciles multiple results from the literature, including the observed scale invariance of center-to-center spacing, as well as the size independence of edge-to-edge spacing.

## Introduction

The fovea (the center of gaze) represents the most exquisite region of the visual field for high-acuity spatial and color vision. It has been well established that visual perception in this area is limited by factors located early in the visual processing stream, such as the physical spacing of retinal cells and the fidelity of the optical signal passing through the anatomical structures in the eye. Elsewhere in the visual field, however, the fundamental limit to visual perception is the enigmatic phenomenon known as “crowding”–the adverse effect of nearby flankers on identification of a target. Whether crowding in the fovea exists, and its relationship to peripheral crowding, has been hotly debated for several decades, and resolving this debate crucially informs models of cortical processing which often rely on the homogeneity of visual cortex across the visual field. Specifically, given the systematic progression of retinal tissue per degree of visual angle of eccentricity^1,2^, it would be most parsimonious if the fovea were merely *quantitatively* different from the periphery, with effects scaling due to the cortical magnification factor^3^. Alternatively, foveal crowding could be *qualitatively* different: it could be “absent,” as some have claimed, or could possibly be an optical effect, rather than a neural effect.

Undoubtedly the most dramatic demonstrations of crowding occur in the peripheral visual field, where the phenomenon is spatially extensive and dominates other performance-limiting factors, such as retinal sampling. Several comprehensive reviews of the effect exist^4–6^. The extent of peripheral crowding has been shown to increase roughly in proportion to the target eccentricity, a relation now known as Bouma’s “Law”^7^. The initial estimate was a peripheral interaction zone of about “half the target eccentricity,” though the exact formulation depends on the stimuli used, and mathematical descriptions are undergoing continual refinement^6,8^. Notably, all formulations of crowding zone extent as a function of eccentricity require precise measurement of the foveal crowding zone with appropriate stimuli.

Some of the earliest investigations of crowding were performed in the fovea of normal and amblyopic observers by Flom and colleagues^9^. They found that the maximal extent of interference from bar flankers on identification of a Landolt C was correlated with the size of the minimum resolvable letter for each subject. An important subsequent finding established that crowding was post-retinal, using an experimental condition where the flankers were presented to one eye and the target to the other, finding equivalent crowding versus non-dichoptic presentations^10^. In a later review, Flom made a precise distinction between the term “contour interaction,” which he used to refer to low-level spatial interactions between nearby contours, and the more general “crowding effect,” which included eye movements and attentional factors^11^. In Flom’s view, the more local and low-level contour interaction can be isolated with a target surrounded by bars, while the more general effect is measured using complex displays comprising multiple letters. More recently, usage of the term “crowding” has been expanded to refer to many interference effects from adjacent flankers–basic low-level spatial interactions, as well as those that likely involve much higher-level influences such as attention. Following modern usage, we use the term “crowding” throughout, even in cases that Flom may have distinguished as “contour integration,” such as our main effect.

While interaction effects from nearby flankers in foveal vision have been previously shown to modulate hyperacuity thresholds in tasks such as vernier discrimination^3,12,13^, results using traditional acuity stimuli such as letter optotypes remain controversial. Foveal crowding with the latter has received some support^9,14–17^ and has been pursued more recently^18–21^. It has been argued that in some of these conditions target and flankers may physically overlap^6^. The deleterious effects in the fovea have also been attributed to simple lateral contrast masking, rather than the characteristic neural spatial disorder that defines crowding^22,23^. Similarly, it has also been proposed that foveal crowding is due to physical properties of the stimuli themselves (i.e., explainable from the spatial frequency content of the targets and neighboring clutter), and independent of neural properties of the visual system, unlike peripheral crowding^24^, though this proposal has been challenged^14,25^.

Importantly, to observe foveal crowding requires procedures to significantly reduce identification performance. The most common method has been to present small, high-contrast stimuli at far distances (physically or optically). To permit testing of larger letter sizes in the fovea, researchers have maintained equivalent performance levels by directly reducing stimulus luminance^18^ or contrast^21^, with contrast reduction implicitly as part of contrast threshold measurement^23^, or by introducing blur^26^. Shortened presentation times (<100 ms) have also been used to observe foveal crowding^19,27^. More recently, Pelli *et al*.^20^ measured interaction effects using a novel font featuring optotypes with an extreme vertical elongation. See Table 1 for a summary of the previous foveal crowding studies. Some studies, using bar or letter flankers with moderate or infinite durations have observed interactions effects, generally until the flankers are on the order of 3–5 arcminutes from the target. On the other hand, crowding in the fovea with has also been reported as “absent” or “weak”^6,28^, particularly with low contrast letters^29,30^. One explanation that has been offered for the previous negative findings concerns the tiny spatial extent of foveal crowding. When previous studies have presented large, low contrast targets and flankers, the target/flanker spacing has typically been specified as a fraction of the target size (at least a bar-width), which in most cases will far exceed the critical spacing. See Siderov *et al*.^21^ for this argument, and a resolution using absolute, rather than nominal, letter spacing. Our goal in this study was to unambiguously identify crowding effects using standard, highly discriminable optotypes presented at the fovea.

**Table 1:** Summary of results from previous studies of foveal crowding.

Study	Maximal spatial extent of crowding: critical spacing (CS)	Maximal crowding	Target stimulus and task	Target size	Flankers	Dur-ation (ms)	Notes
Flom *et al*. 1963	~1.9–3.2′ E-E	0.9–1.3′	Landolt C	2.5′–3.3′	bars	∞	Viewing distance set to yield ~80% unflanked.
Danilova and Bondarko 2006 (Experiment 1)	1.5–4′ E-E	1–2′	Landolt C	2.1–4.7′	bars	500	Viewing distance: 1.7–8.1 m. Critical spacing for asymptotic performance estimated by eye from their Fig. 2.
Danilova and Bondarko 2006 (Experiment 3)	2.5–5′ E-E	0′	Tumbling E	3.5′ and 4.4′	Tumbling Es	500	Viewing distance: 1.7–8.1 m. Critical spacing for asymptotic performance estimated by eye from their Fig. 6.
Strasburger 1991	X	X	Numbers	3.6′	Numbers	100	3.6′ (0.06 deg) was the smallest size tested, with flankers 7.2′ center-center distance. No effect of flankers on threshold contrast.
Wolford and Chambers 1984 (foveal condition)	>6′ E-E	2.4′ (2 of 4 subs.)	Square with gap	12′	lines	17–33	Short duration adjusted to reduce performance.
Siderov, Waugh, Bedell 2013	4′ E-E	1′	Sloan Letters	~4′	bars	∞	high-contrast condition
Toet & Levi 1992	3.6′ C-C	—	T orientation	~1.8–4′	Ts	150	CS: spacing with 75% correct. Target size estimated as (1.2–2.6′) *1.5
Lev *et al*. 2014	>2.8′ E-E	—	E (left or right)	7.2′	E	30–250	Extent not measured, but crowding found at 0.4 character widths
Pelli *et al*. 2016	3.9′ C-C	—	10 numbers, novel font	1.2′ (width)	numbers	∞	Novel font with tall, thin letters. 2–10 m distance.
Present results	0.75–1.3′ E-E	0.5′	Tumbling E	1.85′–4′	Tumbling Es	∞	Conditions for 80–95% performance.

A further consequence of flanking items on target recognition was identified by Flom *et al*.^9^, but has received little direct experimental investigation. Specifically, for bars at the very nearest spacings (within approximately one arc-minute), performance begins to recover, relative to the point of worst performance, which occurred when flankers were about ~40% of the target size from the target^11^. In some cases performance with abutting bars was equivalent or better than performance without bars. This was reminiscent of the earlier results of Takahashi^31^, who found a similar facilitation effect of nearby bars on a meticulous gap detection task in the fovea. Flom^11^ proposed that this effect was due to optical spreading of the image on the retina. Subsequent investigations of foveal contour interaction or crowding have had mixed results revealing this aspect of flanker interactions. For many studies, this effect is not explicitly mentioned, but can be inferred from raw data plots. See Table 1 for a summary; a non-zero entry in the ‘maximal crowding’ column indicates a recovery region. It is ubiquitous with basic stimuli such as Landolt-Cs flanked by bars^9,14^, but has also been observed with Sloan letters flanked by bars^18,21^, exhibiting a dependence on the spatial characteristics of targets and flankers.

The major obstacle to definitive results concerning foveal spatial vision is the presence of optical aberrations. Irregularities in the cornea and lens of the eye blur the light falling on the retina and constitute pre-neural limits to fine spatial vision. Accordingly, these characteristics determine the smallest stimuli that can be delivered to the retina and resolved in isolation. Therefore, any measure of spatial vision at the fovea necessarily confounds optical and neural factors. Adaptive optics (AO) offers an opportunity to bypass the effect of ocular imperfections and deliver near diffraction-limited stimuli to the eye unhindered by monochromatic aberrations. While its primary application has been to improve the spatial resolution of retinal images, AO has also been used to measure the visual benefit of correcting optical aberration on a variety of psychophysical tasks – acuity and contrast sensitivity in normal observers^32,33^, real-life tasks^34^, color vision^35^, as well as visual performance after correction of highly aberrated keratoconic eyes^36^. For a detailed review on the use of AO for studying visual function, see the review by Roorda^37^. Concomitant imaging and stimulus delivery via AO correction has allowed relating the neural architecture of the retina to visual perception^38–42^. Here, we used a custom AO-based vision testing setup to measure the limits of crowding at the fovea. Under these conditions, tailored to reveal minute spatial interactions, we sought to identify whether crowding exists in the fovea and, if so, to quantify its extent on a precise scale.

## Results

### Representative data and model

**Figure 1:**
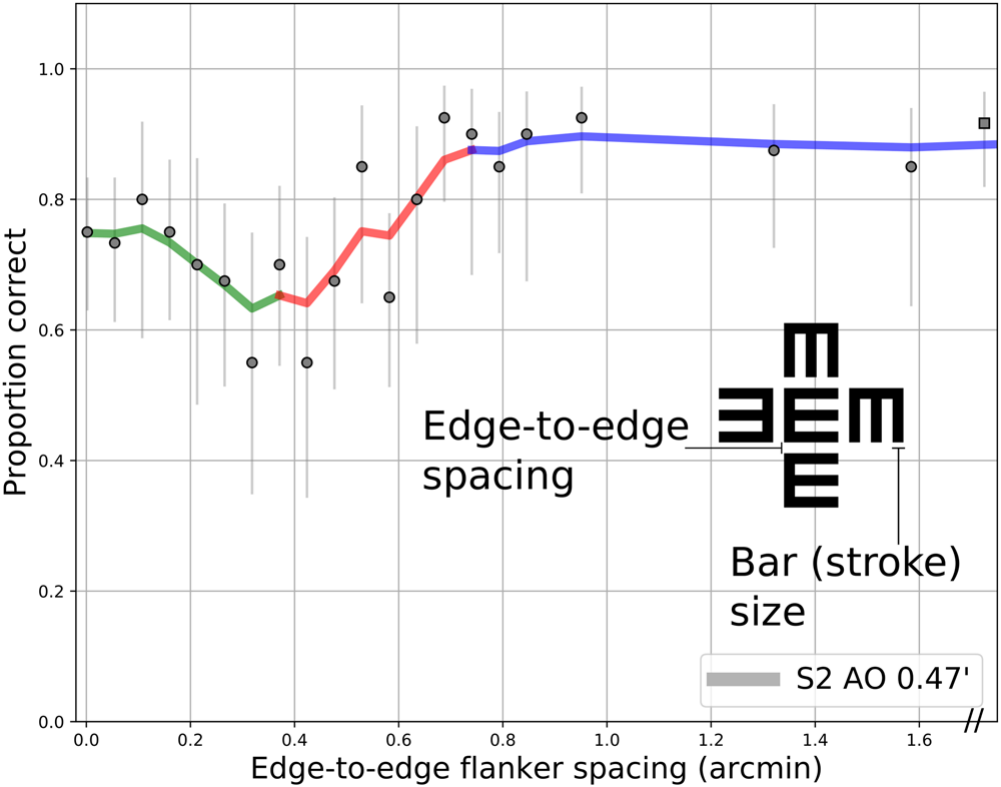
Representative data for subject S2 with adaptive optics correction, at a bar size corresponding to an approximately 20/10 letter. Proportion correct is plotted against the size of the intervening gap (in arcminutes). The curve shows non-parametric smoothing, colored to demonstrate the three distinct regions defined in the text. Error bars indicate 95% confidence intervals from Bayesian estimation of proportions. Square marker shows unflanked trials.

Proportion correct was fit individually for each subject, optotype size, and optical condition, yielding a set of performance-vs.-flanker spacing curves. Representative data are shown in Fig. 1 for observer S2, with adaptive optics correction and a Tumbling-E letter with a bars subtending 0.47 arcminutes. The solid data points indicate average performance at each spacing, with error bars representing 95% confidence intervals from maximum likelihood estimation based on the raw data and binomial statistics. The line shows a non-parametric smoothed curve through the datapoints. The curve can be divided into three regions, depicted with the colored segments in Fig. 1. For sufficiently large spacings (rightmost section of curve, in blue), performance is asymptotic at a high performance level, which corresponds to the unflanked proportion correct. With flankers located closer to the target (middle section of curve, in red), flankers reduce performance, in typical fashion for crowding experiments. The farthest flanker spacing at which performance worsens below the asymptotic level is known as the *critical spacing*, here approximately 0.75 arcminutes. The magnitude of reduction that defines the transition is not agreed upon in the literature. Some researchers have estimated the maximum spacing causing interference by eye^9,18,21^, others have utilized a criteria of statistical significance^14^, while others have interpolated the critical spacing from a parametric fit, with an amplitude reduction by a factor of 1/e^43^, or, alternatively, by an amount of 10%^44^. Here, to identify the largest flanker spacing that could exhibit interference, we adopt a conservative criteria of a reduction of 0.025 from the fitted asymptotic performance level. In accordance with previous literature, performance was modeled with a cumulative Gaussian function, with free parameters for the mean (horizontal position, or point of subjective equality) and standard deviation (“slope” or width). The curve was corrected for the 25% guess rate and multiplicatively scaled with an amplitude term corresponding to the asymptotic performance level.

With flankers very close to the target, in many cases performance was improved relative to the location of maximal crowding (approximately 0.35 arcminutes for this example). This part of the curve is called the recovery region (green section), and was modeled as a linear relationship of performance versus flanker spacing, with a negative slope. In total, performance vs. flanker spacing curves are defined as the numerical *maximum* of the sigmoidal (crowded plus asymptotic) and linear recovery portions.

The relations are stated formally as Equations 1a, 1b, and 1c below. The recovery portion (Equation 1b) is simply the straight line parameterized as mx + b, with edge-to-edge spacing “x” as the abscissa. The sigmoidal portion (Equation 1c) consists of a cumulative Gaussian with the standard width and location parameters σ and μ, respectively. Parameter A scales the cumulative Gaussian and denotes the asymptotic level, with the sigmoidal curve corrected for chance based on the guess rate gamma (fixed at 0.25 for the Tumbling-E task). Each curve was fit individually with this five-parameter model using Bayesian estimation to determine the most likely parameters and their resultant 95% confidence intervals. Fitting was performed with Python and the NumPy/SciPy tools^45^, using the PyMC3 library^46^ for Markov chain Monte Carlo estimation. Weak uniform prior distributions were used for all parameters as follows: A (0:1.0), σ (0:5), μ (−1:1.0), m (−1:0), and b (0:1.0).

1a $${\Psi }(x|m,b,A,\mu ,\sigma )=max({{\Psi }}_{recovery}(x,m,b),{{\Psi }}_{crowding}(x,A,\mu ,\sigma ))$$

1b $${{\Psi }}_{recovery}(x|m,b)=mx+b$$

1c $${{\Psi }}_{crowding}(x|A,\mu ,\sigma )=\gamma +(A-\gamma )\ast \Phi (x,\mu ,\sigma )[\gamma =0.25]$$

To construct a general model that might account for variability across observer, stimulus size, and optical condition, we first normalized each psychometric function to their asymptotic level, yielding a scale reflecting the performance reduction relative to unflanked targets. Furthermore, for results measured on a proportion correct scale, identical changes in performance are not equivalent across the psychometric function, which is nonlinear. For example, a drop in identification accuracy from 95% to 90% represents a more significant effect than a change from 65% to 60%. To compensate for this nonlinearity, Z-scores (or “probit units”), the inverse transformation of the cumulative Gaussian distribution, were used, resulting in a unit akin to d-prime or sensitivity. To fit the data using this scheme, the ordinate was converted to a relative scale indicating the change in Z-score units from the fitted asymptotic performance level. For example, an ordinate of 0 on this scale results from spacings achieving asymptotic performance levels, and negative numbers indicate a drop from this level (in Z-score units). For the examples above, a reduction from 95% to 90% corresponds to a change in (1.645–1.282) = 0.363 Z-units, whereas the reduction from 65% to 60% corresponds to (0.385–0.253) = 0.132 Z-units. Proportions were corrected for guessing prior to Z-score computation.

**Figure 2:**
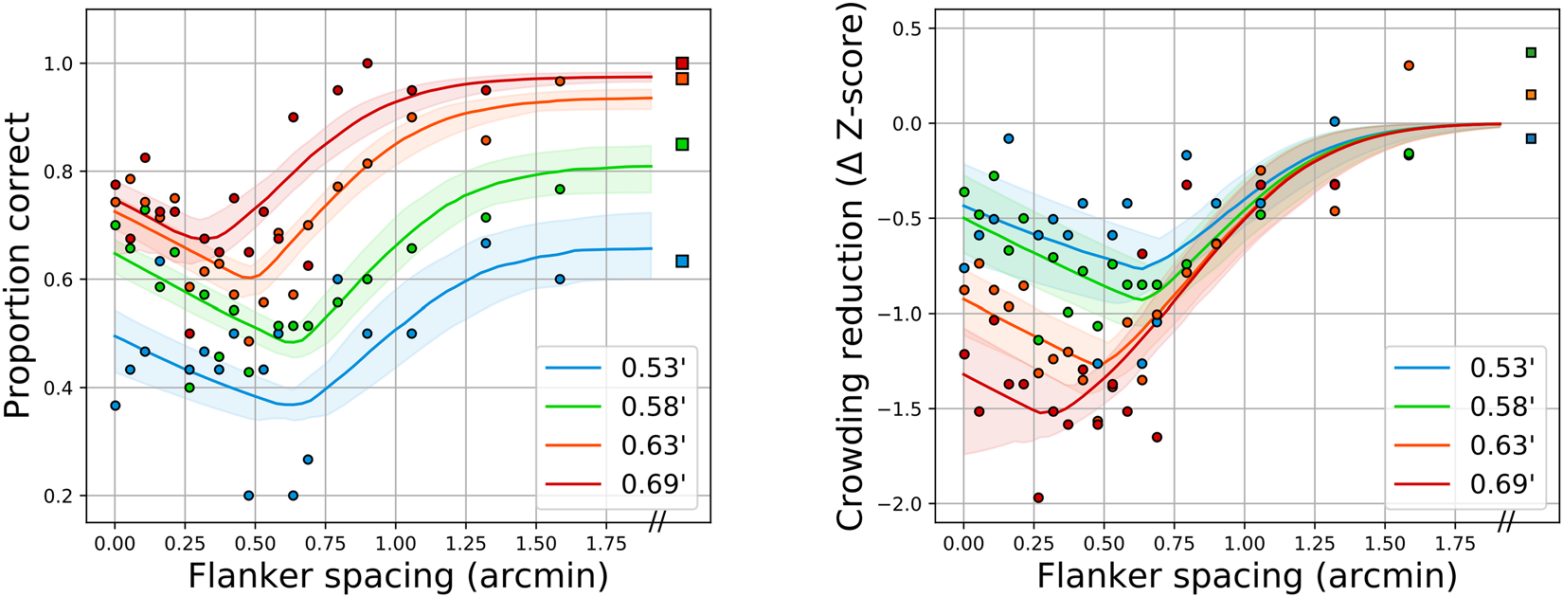
Representative performance-vs.-spacing curves for subject S3 with AO. Left panel: raw proportion correct as a function of flanker spacing (in arcminutes), right panel: Z-score-normalized model, with reduction in Z-units from asymptote as the ordinate. The four letter sizes tested are indicated in the legend in terms of the size of a single stroke.

Figure 2 shows an example of this transformation, with all stimulus sizes for subject S3 with AO, shown as raw proportion correct (left panel) and reduction from the uncrowded level in terms of Z-scores (right panel). Lines indicate best-fitting model and 95% confidence intervals for the parametric fits. Remarkably, this normalization aligns the crowding-related segments of the curves, across all sizes for a given observer and condition, suggesting that the width and location parameters of the sigmoidal portion may be invariant across stimulus size. The recovery portion differs between sizes, however, evident by the non-alignment of the diagonal colored limbs at the closest spacings—they are approximately parallel, but stacked vertically. To determine which model parameters should be fixed or free across conditions and observers, we performed Bayesian model comparison on the set of possible models, described below.

### Fitted parameters and model comparison

**Figure 3:**
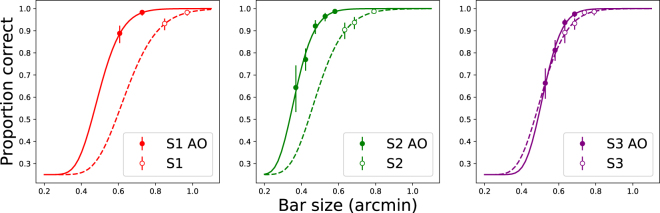
Unflanked performance for each subject in each condition, as indicated in legend. Letter size is indicated on the x-axis, corresponding to the width of a single stroke. Lines show best-fitting cumulative Gaussian functions describing proportion correct as a function of bar size (for display only, not used in subsequent analysis).

First, the asymptote parameter A identifies the unflanked performance level at each size and condition for each observer. These are plotted in Fig. 3. The lines show cumulative Gaussian functions fitted to the estimated asymptotes. The performance improvement facilitated by adaptive optics for each subject is evident as the leftward shift, equivalent to MAR improvements of 0.2, 0.225, and 0.025 arcminutes for each subject (respectively). Table 2 indicates MAR at a 75% performance level.

**Table 2:** Participant characteristics, unflanked performance levels, and stimulus sizes tested.

Subject	Habitual spherical correction	Sizes tested with AO (arcmin. of one stroke; 1/5 of letter size)	Estimated 75% Acuity w/AO	Sizes tested w/o AO (arcmin. of one stroke)	Estimated 75% Acuity w/o AO
S1	4.5D	0.61, 0.73	20/11 (0.55′ MAR)	0.85, 0.97	20/15 (0.75′ MAR)
S2	—	0.37, 0.42, 0.47, 0.53, 0.58	20/8 (0.4′ MAR)	0.63, 0.69, 0.79	20/12.5 (0.625′ MAR)
S3	1.5D	0.53, 0.58, 0.63, 0.69	20/12 (0.6′ MAR)	0.63, 0.69, 0.74, 0.79	20/12.5 (0.625′ MAR)

S2 is an emmetrope.

**Figure 4:**
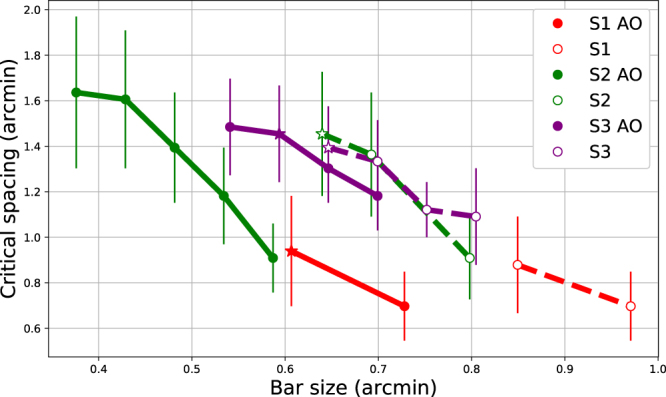
Edge-to-edge critical spacing for each subject and optical condition. Ordinate indicates flanker spacing at which performance drops 2.5% from asymptotic/unflanked performance. Abscissa indicates the stroke width for that condition. Error bars show 95% confidence intervals resulting from 1000 Monte Carlo-generated psychometric functions. Stars indicate conditions at which unflanked performance was 80–95% correct.

Fitted critical spacings are plotted in Fig. 4. Within a subject and optical condition, the critical spacing is largest with the smallest letters, and decreases as letters increase in size. There is no systematic pattern across the entire dataset, indicating that individual differences play a role in determining the critical spacing, rather than the zone being a fixed function of bar size. Conditions with unflanked performance at 80–95% are indicated with stars, for comparison to standard practice in the literature. For these conditions, critical spacings were 0.75–1.3 minutes.

Strictly speaking, only two parameters were required to change with each stimulus size: the asymptote (*A*) and the recovery asymptote (*b*). The remaining three parameters (σ, μ, and m) could be constrained in several possible ways. First, each parameter could vary independently with observer and size, yielding the full/saturated model with many free parameters. Alternatively, parameters could be fixed across sizes, as suggested by the observed alignment of the crowding portion. Furthermore, parameters could be either fixed within an observer for both optical conditions (AO and non-AO) together, or independently for the two conditions. Finally, parameters could be completely yoked together for all observers and conditions.

The results of evaluation of 12 models are given in Table 3. Models ranged from the most constrained model where the three parameters are each yoked together for all observers, sizes, and optical conditions (model #0), to the least constrained/most saturated version of complete independence (model #11), which was used earlier. Goodness of fit was quantified using WAIC^47^, a fully Bayesian fitness measure like the more well-known chi-squared, AIC/BIC and the newer deviance information criteria (DIC), except that WAIC uses the entire model posterior distribution, resulting in more accurate estimates^48^. Like other fitness measures, models with more parameters are penalized, in order to combat overfitting. Lower scores reveal better-fitting models. The effective number of parameters (pWAIC) can be fractional, as the estimate is not simply an integral count of free parameters. Fitting was performed using PyMC3, with 1000 estimates generated for each model tested.

**Table 3:** Model comparison results.

Model #	σ	μ	m	WAIC	pWAIC	LogLik
*5*	O	O	F	4054.7	46.12	2085.77
*6*	O	O	O	4056.59	46.38	2090.85
*1*	F	O	F	4064	48.34	2083.9
*8*	C	C	F	4064.54	53.73	2099.41
*7*	F	C	F	4066.14	52.56	2087.52
*10*	N	O	F	4071.1	55.58	2111.92
*9*	C	C	C	4075.29	67.44	2127.68
*2*	O	F	F	4088.33	45.97	2100.38
*3*	O	F	O	4090.84	47.82	2104.61
*11*	N	N	N	4091.7	73.62	2205.4
*0*	F	F	F	4100.69	50.07	2098.27
*4*	F	F	O	4101.85	53.75	2100.92

Models are ordered from best-fitting (lowest WAIC) to worst-fitting (highest WAIC). Effective number of parameters are given by pWAIC, and the log-likelihood of each model is given by LogLik. WAIC relates to the likelihood, minus a parameter penalty. Models reflect hierarchical constraints on the three parameters, described in the text.

In Table 3, models are ordered by increasing WAIC. The degrees of freedom accorded to the variables in each model are indicated with several codes: “N” (no constraints: freely varies), “O” (fixed for each observer across sizes), “C” (fixed for each observer across sizes, separately for the two optical conditions), and “F” (fixed across observers). Two models were clearly superior: models #5 and #6, in which the two crowding sigmoid parameters (width and location) were fixed for each observer. The location parameter was most critical; models that attempted to fix this parameter across observers comprised four of the worst five models tested. The recovery slope could be yoked across all three observers (#5), or fit separately for each (#6), with only a slight preference for the yoked model (#5). In general, models with individual sigmoid location parameter for each observer were generally best (#5, #6, #1), and separating the optical conditions (#8 and #9) tended to not improve fitness. Models with the most degrees of freedom (#9, #10, #11) were typically worse, despite fitting the data better (higher log-likelihood).

**Figure 5:**
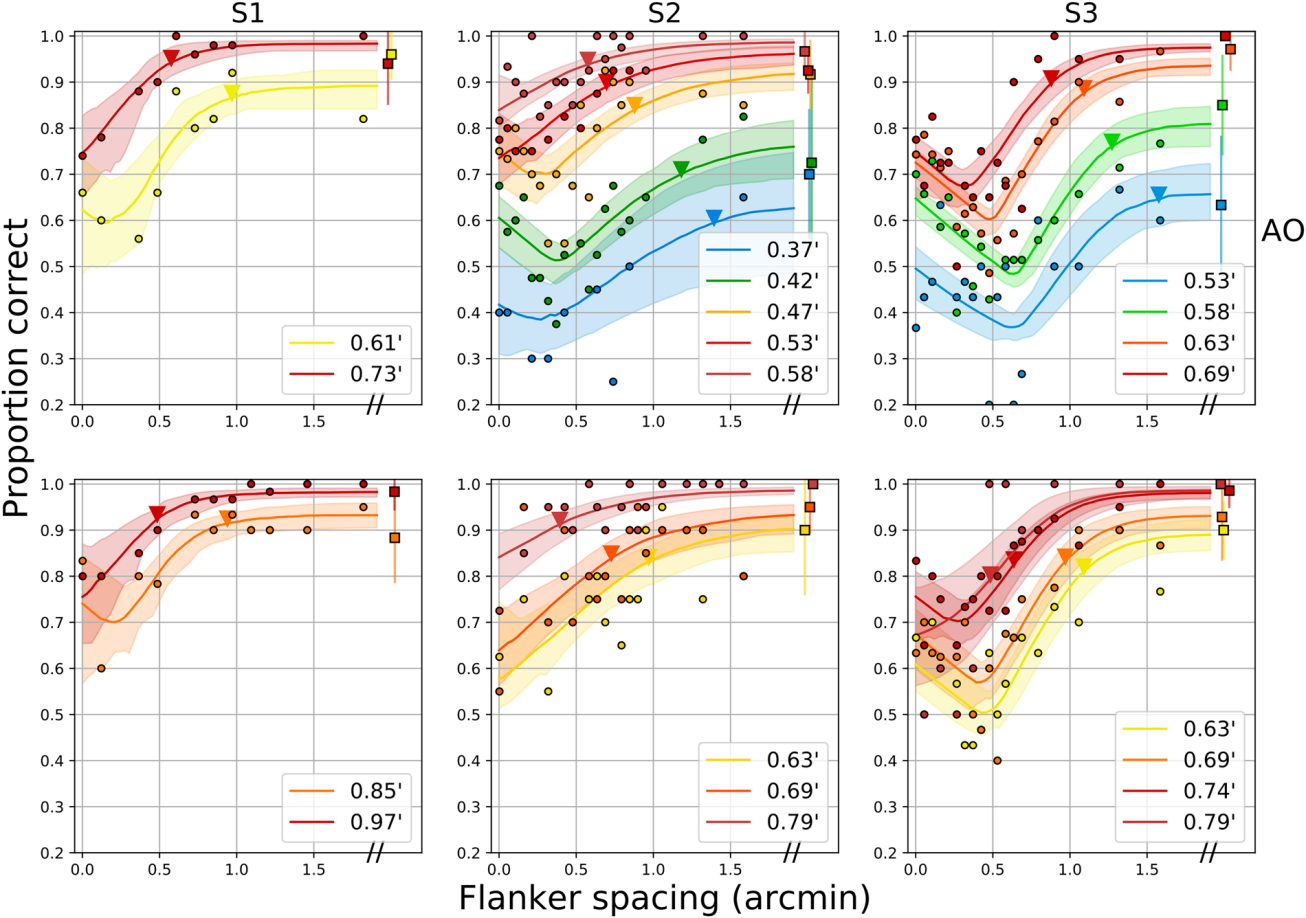
Entire data set for the 3 observers, with fits from model #5. The top row shows AO results, while the bottom row shows low-order optical correction. 95% confidence intervals are shown for unflanked trials (rightmost squares), representative of confidence intervals for the other data points. Colors indicate the size of a stroke, as indicated in the figure legends. Lines and shaded regions show model fits and 95% confidence intervals for each optotype size, which have been inverse transformed from the probit model. Triangles near each function indicate the fitted critical spacings for a reduction of 2.5% accuracy performance.

With the best models (model #5), the Gaussian parameters were fixed across all sizes for each observer (as suggested by Fig. 2), meaning that the results for all sizes of each observer can be captured by a single canonical crowding function. Figure 5 shows the entire data set fit with this model, with critical spacing estimates indicated by the triangle along each function.

## Discussion and Conclusions

In summary, we have shown that the fovea is not immune to crowding, in a standard spatial vision paradigm (high-contrast, unlimited duration Tumbling-E optotypes) with clearly non-overlapping flankers, confirming the neural basis of the phenomenon seen with traditional means^9,14,18,21^. Whereas previous studies have typically reduced the visibility of targets, either by reducing their contrast, luminance or duration in order to observe interference effects, we instead used adaptive optics to counteract the natural blur impeding normal spatial vision in the fovea, permitting the presentation of tiny optotypes and precisely-sized gaps with greater precision than previously possible.

The critical spacings we observed were smaller than those previously found for letter acuity stimuli (see Table 1). In the regime of most crowding experiments, where unflanked levels are held at 80–95%, the critical spacings we observed were 0.75 to 1.3 arcminutes edge-to-edge (or 3.0–6.0 arcminutes center-to-center, depending on the subject and condition). These are much smaller than the 4–5 arcminute spacings that have typically been found in the literature^9,14,18,21^. Even without adaptive optics correction, we measured critical spacings one quarter to one half of previously-reported extents. There are several possible causes for the difference, including our use of a Maxwellian view adaptive optics setup, with an artificial pupil and a bite bar to control fixation. Fixation stability may have played a role, as Bedell *et al*.^49^ found that imprecise eye movements may contribute to increased difficulty correctly locating letters in a string, as previously proposed by Flom^11^. While Bedell *et al*.^18^ found minimal effects of luminance on foveal crowding extent, optical factors may have played a role. Lastly, our use of Tumbling E stimuli may have contributed to smaller critical spacings. However, Danilova and Bondarko^14^ found critical spacings for Tumbling Es flanked by Tumbling Es of approximately 2.5–5 arcminutes: twice as extensive as our results.

When proportion correct was converted to reduction in Z-score units from the asymptotic (unflanked) performance level, a single canonical crowding curve emerged for each subject that could account for all stimulus sizes, with both AO correction and natural viewing. With this transformation, flankers had a maximum influence zone of 1–1.5 arcminute (edge-to-edge), reducing performance by about 1 Z-score unit for each half arcminute of flanker proximity. As mentioned previously, the standard practice has been to test crowding with specific sizes to avoid floor and ceiling effects. Here we directly model the relationship between target size and foveal crowding extent, capturing the fact that crowding functions flatten (in terms of proportion correct) for stimuli that are more difficult to resolve. The slope is constant when specified in terms of a reduction in Z-score. Hence, flankers multiplicatively affect identification performance in a consistent manner.

The recovery portion of the curve likely reflects a different mechanism. This effect has been observed previously in the literature^9,14,21,31^, but as far as we know has not been explicitly characterized. Here this effect is robust, though absent for conditions with very high asymptotic performance levels (largest letter for each subject), and more variable without AO. Targets with abutting flankers were identified with an accuracy approximately 20% lower than unflanked targets. Performance worsened with increasing flankers separation, until overtaken by the typical crowding effect (sigmoidal function of flanker spacing) at spacings of approximately 0.5–0.75 minutes. This transition occurs at a spacing one-half to one-quarter of that seen in previous studies. Previous authors have proposed that the recovery effect results from “optical smearing” of flanking bars with the target^11^, which implicates spatial cues that would be most useful for bar flankers and simple targets such as Tumbling Es or Landolt Cs. Takahashi^31^ observed this influence on gap detection even with wide bars, finding that only the nearest edge defined the resultant curve, which included a significant recovery portion. In another study, thinner bars were found to exhibit some recovery, although the effect was most robust with bars matched to the size of the target’s bars^14^. We believe that in our paradigm, the nearest limbs of the flanking Es behave much like bar flankers. The spatial cues that result from the integration of the flanking bars with the target cause a noticeable widening of the target bars on each side except its opening (to the right for an upright E), a subtle low-level cue that subjects quickly learn. It has been found earlier that subjects can use width cues for spatial judgments, even when two adjacent lines are within the resolution limit^50^. Though most evident with Tumbling Es or Landolt Cs, a similar recovery pattern has also been observed with high-contrast Sloan letters^18,21^, again likely resulting from spatial cues inferred by acute observers. Importantly, in our study optical influences were carefully controlled yet the recovery persisted, contrary to Flom’s proposal for a purely optical contribution^11^. This suggests that *neural* smearing of the target and flanker features is the probable cause of this component, with a spatial extent much smaller than the interference effect. A possible mechanism is spatial summation, which was recently shown to be post-receptoral using adaptive optics and eye-tracking^51^.

Our results differ from those found in peripheral vision in several ways. First, critical spacings were parsimoniously captured by edge-to-edge spacing, rather than center-to-center spacing. We believe that this property may be unique to foveal vision^22^. Second, the robust flanker recovery, while common in foveal contour interaction studies, is generally absent in peripheral experiments, though it was seen in the parafovea (0.54 and 1.05 degree) in the flanked gap-detection task of Takahashi^31^ and with very brief peripheral presentations (2 or 5 degrees) of a square with a gap on one side, flanked by lines^27^. Lastly, several studies have reported that the center-to-center critical spacing in the periphery is independent of stimulus size. Importantly, these studies adjusted the contrast of differently-sized stimuli to test at similar performance levels, either by explicit manipulation of stimulus parameters^43^, or as a result of a contrast threshold procedure^52,53^. Our finding that critical spacing depends crucially on asymptotic performance agrees with these findings, though with edge-to-edge critical spacing in the fovea–in agreement with Siderov *et al*.^21^.

**Figure 6:**
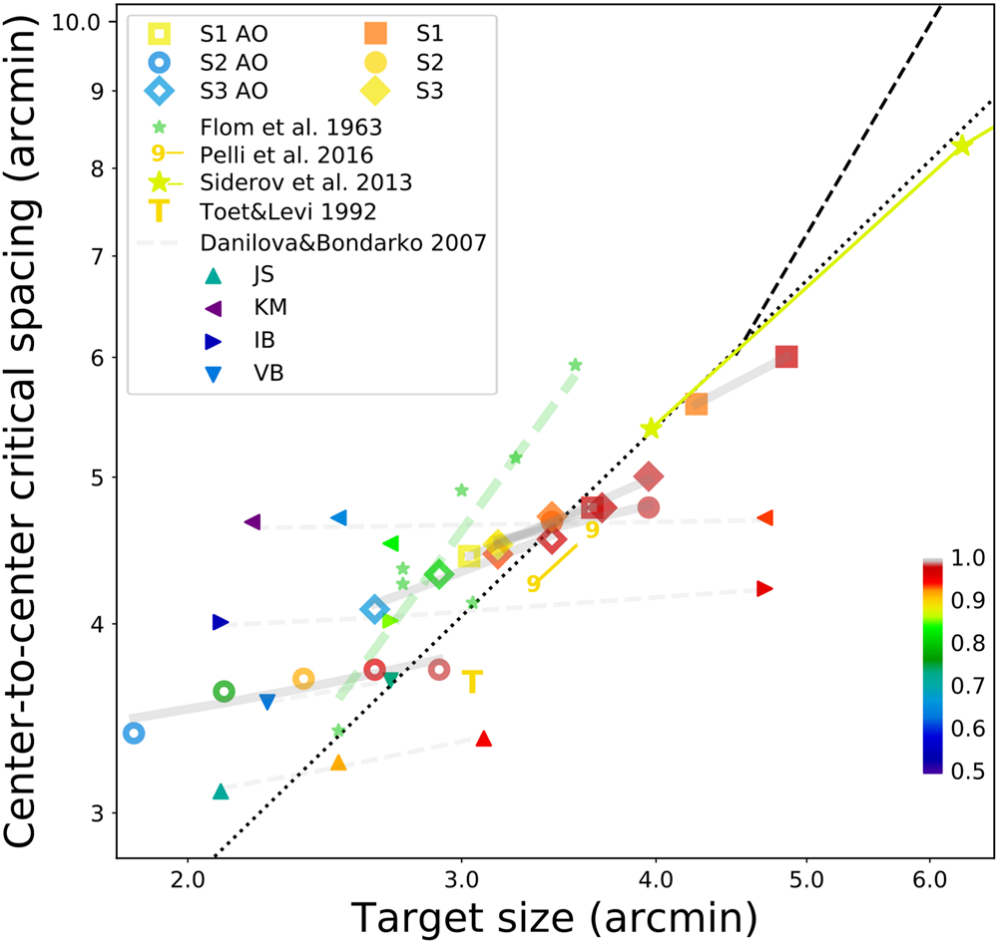
Summary figure plotting center-to-center critical spacing versus target size for our results and relevant results from the literature. Each marker type represents a study or individual observer. The color of each symbol indicates the asymptotic (unflanked) performance level, as shown by the colorbar. Gray and colored lines connect results from a single study (or observer from a study). Black dotted line indicates proportional regime (CS = 1.4 × size) for ~90% correct).

Reconciling the spatial extent of crowding across studies in foveal and peripheral vision has proven to be elusive. One approach is to plot center-to-center critical spacing versus target size, allowing comparisons of critical spacings across varying stimulus conditions^22,26,54,55^. This analysis has proven useful in showing how peripheral, crowding-limited data follow one pattern and foveal, blur-limited data follow another pattern^26,55^. Figure 6 extends this analysis more precisely into the fovea, plotting our own data (fitted with model #5) and those from several studies from Table 1. The dotted line is based on foveal results from contrast thresholds of blurred stimuli, revealing a “size-limited” regime, which also matches results from non-strabismic amblyopia^26^, non-foveal AMD patients^55^ and S-cone isolating stimuli^56^. For results on this line, critical spacing is proportional to stimulus size, such that critical spacing = 1.4 × size, a straight line parallel to the identity line on this log-log plot. The peripheral crowding line is not relevant for this discussion, but is shown for reference as the dashed line in the upper part of the plot. Data in the periphery (from 3–10 degrees) has been shown to fall on this line^26,55^, where the critical spacing rises steeply compared to the threshold size.

Since the ordinate of this plot requires center-to-center spacing, we first converted data into center-to-center measures. For targets flanked by bars, this value is the edge-to-edge spacing plus 0.6 times the target size. For letters flanked by letters, center-to-center spacing is simply the edge-to-edge spacing plus the letter size. We have colored each symbol on the figure according to the corresponding asymptotic performance level, with values determined from published plots^14,21^ or as stated in the texts^9,17,20^. As mentioned previously, to allow the same criteria for critical spacings that we used (the abscissa for approximately asymptotic performance), we estimated critical spacings by eye from raw data plots when possible^14,21^. The two prior studies with the smallest target sizes and critical spacings both utilized Landolt C stimuli flanked by bars^9,14^. Flom *et al*.^9^ controlled the distance such that each observer achieved approximately 80% unflanked, and each observer is shown, with all observers connected with the green dashed line. Danilova and Bondarko^14^ tested several observers at several asymptotic performance levels. Here we connected each observer’s conditions with a gray dashed line. Two other studies used non-standard stimuli at a single high performance level: Tumbling T’s flanked by T’s^17^ and a novel elongated number font^20^. Lastly, Siderov *et al*.^21^ tested Sloan letters flanked by bars, at an asymptotic level of approximately 90% correct.

The colorization reveals a remarkable patterns in the results. Individual results at the same performance level form parallel diagonal lines on this plot, parallel to the dotted line. For example, the individual observers from Flom *et al*.^9^ and the appropriate conditions of Danilova and Bondarko^14^ fall approximately on a single line for the ~80% performance level (greenish color), above and to the left of the dotted line. Asymptotic results closer to 90% were used by Toet and Levi^17^, Siderov *et al*.^21^, and would be expected from the adaptive procedure used by Song *et al*.^26^ and Pelli *et al*.^20^ These are indicated in yellow, on or near the dotted line. Multiple conditions of Siderov *et al*.^21^ are all on this same line. Although their conditions correspond to different stimulus sizes, they lowered the contrast to maintain equivalent visibility and asymptotic performance, and are therefore consistent with the pattern we report. Finally, both our data and those of Danilova and Bondarko^14^ show that center-to-center critical spacing is fairly flat within each observer measured at different target sizes (but with different asymptotic levels), with a slight increase as target sizes increase. The steeper slope for our data may be the result of either our Tumbling-E targets or usage of letter flankers rather than bar flankers.

There are several conclusions to take away from this analysis. First, the good fit of these data on lines parallel to the dotted line indicate that, in the fovea, the center-to-center crowding zone is proportional to the stimulus size for a wide range of stimulus conditions. That was proposed initially by Flom *et al*.^9^, although they based their arguments on the edge-to-edge spacing of several individual observers including amblyopes. Second, as noted by Siderov *et al*.^21^, edge-to-edge spacing is consistent across stimuli sizes, but here we clarify that maintaining equivalent performance levels (which they achieved with contrast) is crucial. The equal edge-to-edge spacings mean that center-to-center spacing is proportional, also in agreement with Song *et al*.^26^, and Levi *et al*.^23^. Finally, for situations with different target sizes at different asymptotic levels (Danilova and Bondarko^14^ and our study), there are three observations. First, the edge-to-edge spacing of the crowding zone decreases for larger targets (Fig. 4), which is why crowding “disappears” for sufficiently well-resolved foveal targets. On the other hand, the center-to-center extent of the crowding zone shows a slight increase as a function of size, shown by the positive slope of individual observer lines in Fig. 6. Finally, if measured as a normalized Z-score reduction from asymptotic performance (Fig. 2), edge-to-edge critical spacings are exactly the same across sizes, which would result, again, in proportional increases for center-to-center spacing.

As theories attempt to link the crowding zone size to anatomical measures such as the extent of lateral neural connections or receptive field sizes^4–6^, it is vital to accurately characterize the extent of crowding at the fovea. It is straightforward to *increase* the size of the crowding zone, by reducing stimulus visibility such as with shortened stimulus duration^43,44,57,58^ or by decreasing contrast^59,60^. However, these manipulations introduce possible confounding effects to determination of the actual extent of the crowding zone. Instead, our method allowed testing of standard, high-contrast, long duration acuity targets smaller than those previously possible with traditional methods. Thus, these results provide the most accurate estimate of the spatial extent of crowding at the fovea, free of pre-neural limiting factors that may have obscured previous measures.

## Methods

### Adaptive optics vision simulator

**Figure 7:**
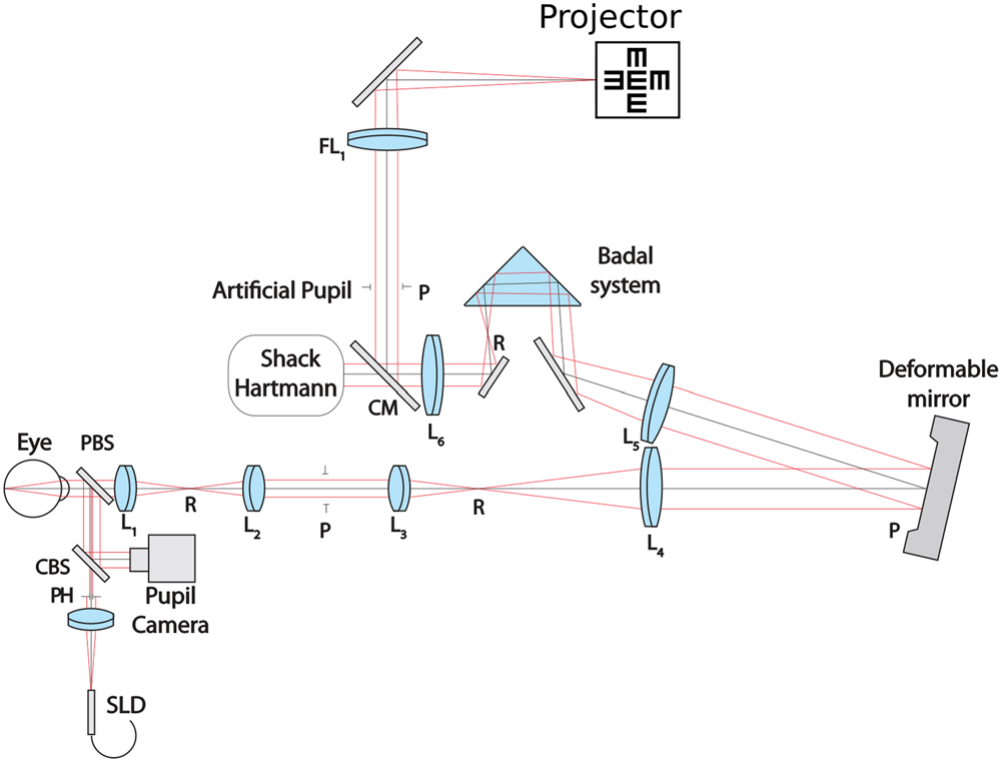
Schematic of the optical apparatus.

The apparatus was a custom-built optical system consisting of a deformable mirror (Mirao52D. Imagine Eyes, Orsay, France) and a wavefront sensor as its key components. A superluminescent diode centered at 840 nm served as the wavefront sensing beacon. The light source was collimated and passed through an 800 micron pinhole en route to the eye. This ensured that the effect of aberrations on the beacon during the first pass into the eye was minimal. A 90:10 (Transmission:reflection) pellicle beamsplitter reflected the wavefront sensing beacon into the eye. The light scattered back from the retina was imaged on to the wavefront sensor. Image relay optics were constructed using a train of achromatic lenses to relay the scattered light from the eye’s pupil onto the deformable mirror and the lenslet array of the wavefront sensor. An extra pupil conjugate plane was constructed to allow insertion of trial lenses to compensate for sphere and cylinder. The wavefront sensing arm of the setup was separated from the vision testing arm using a cold mirror. An artificially adjustable iris allowed for selecting the optimum pupil size for vision testing. The size of the field on the retina was 1 deg. Stimuli were displayed on a computer projector (Sharp PG-M20X) placed at an imaging plane and the retinal illuminance through a 6-mm eye’s pupil was measured to be 100 cd/m^2^. Aberrations were corrected dynamically in a closed-loop fashion over a 6.75 mm eye’s pupil while vision testing was performed over an artificial 6-mm pupil. A continuous AO correction allowed stable optical quality throughout vision testing. Subjects were allowed to blink at their discretion during psychophysics. Prior to vision testing, the subjects were subjectively refracted to compensate for the longitudinal chromatic aberration arising between the wavefront sensing beacon and the stimulus display. A schematic of the system is shown in Fig. 7.

### Stimuli and Procedure

The stimuli were high contrast black-on-white Tumbling-E targets, presented in isolation or flanked by four randomly-oriented Tumbling-Es located above, below, and to either size side of the target. Stimuli were displayed until the subject responded. Blocks comprised 300 trials, with several combinations of sizes and spacings interleaved to yield 10 trials per size/spacing condition. Sizes were chosen to yield a range of reasonable performance levels along the psychometric function for each observer, with spacings ranging from 1.6 arc minutes of space between target and flanker, to abutting (no space between target and flanker), and an unflanked condition. Each size and spacing was evaluated in at least two separate sessions. The wavefront measurement and closed-loop correction were continuously monitored, with appropriate adjustments performed as necessary within each trial. Subjects were encouraged to take breaks whenever needed between trials. A condition without AO correction was also run wherein subjects’ sphere and cylinder were minimized using trial lenses.

### Participants

The University of Washington institutional review board approved this research and all subjects signed an informed consent before their participation in this study. All procedures involving human subjects were in accordance with the tenets of the Declaration of Helsinki. Three subjects participated in the study. All were authors, with one subject/author (S3) naive to the purpose of the study during the experiment. S2 was emmetropic and an experienced observer in this apparatus, while S1 was an experienced psychophysical observer. S1 and S3 were myopes and their prescription was corrected with trial lenses. Accommodation was paralyzed with 1% tropicamide ophthalmic drops and a bite bar was used to minimize head movements. Table 2 summarizes participant characteristics and basic stimulus parameters.

### Data Availability

The datasets generated during and/or analysed during the current study are available from the corresponding author on reasonable request.
